# HEPA filters of portable air cleaners as a tool for the surveillance of SARS‐CoV‐2

**DOI:** 10.1111/ina.13109

**Published:** 2022-09-18

**Authors:** Isabel G. Fernández de Mera, Carmen Granda, Florentina Villanueva, Marta Sánchez‐Sánchez, Alberto Moraga‐Fernández, Christian Gortázar, José de la Fuente

**Affiliations:** ^1^ SaBio, Instituto de Investigación en Recursos Cinegéticos IREC (UCLM‐CSIC‐JCCM) Ronda de Toledo Ciudad Real Spain; ^2^ Residencias CADIG Guadiana I y II Centro de Salud Ciudad Real I Spain; ^3^ Instituto de Investigación en Combustión y Contaminación Atmosférica Universidad de Castilla‐La Mancha Ciudad Real Spain; ^4^ Parque Científico y Tecnológico de Castilla‐La Mancha Paseo de la Innovación 1 Albacete Spain; ^5^ Department of Veterinary Pathobiology, Center for Veterinary Health Sciences Oklahoma State University Stillwater Oklahoma USA

**Keywords:** aerosols, HEPA filter, indoor air, portable air cleaners, SARS‐CoV‐2, surveillance

## Abstract

Studies about the identification of SARS‐CoV‐2 in indoor aerosols have been conducted in hospital patient rooms and to a lesser extent in nonhealthcare environments. In these studies, people were already infected with SARS‐CoV‐2. However, in the present study, we investigated the presence of SARS‐CoV‐2 in HEPA filters housed in portable air cleaners (PACs) located in places with apparently healthy people to prevent possible outbreaks. A method for detecting the presence of SARS‐CoV‐2 RNA in HEPA filters was developed and validated. The study was conducted for 13 weeks in three indoor environments: school, nursery, and a household of a social health center, all in Ciudad Real, Spain. The environmental monitoring of the presence of SARS‐CoV‐2 was conducted in HEPA filters and other surfaces of these indoor spaces for a selective screening in asymptomatic population groups. The objective was to limit outbreaks at an early stage. One HEPA filter tested positive in the social health center. After analysis by RT‐PCR of SARS‐CoV‐2 in residents and healthcare workers, one worker tested positive. Therefore, this study provides direct evidence of virus‐containing aerosols trapped in HEPA filters and the possibility of using these PACs for environmental monitoring of SARS‐CoV‐2 while they remove airborne aerosols and trap the virus.

**PRACTICAL IMPLICATIONS**

- A method for detecting SARS‐CoV‐2 RNA in HEPA filters was developed and validated.
- HEPA filters tested positive for SARS‐CoV‐2 in two school classrooms despite the mandatory use of masks.
- Direct evidence of virus‐containing aerosols trapped in HEPA filter is provided.
- The school and nursey classrooms studied were negative for SARS‐COV‐2 RNA, both for the HEPA filter and surfaces, consequently COVID‐19 cases were not notified.
- The positive HEPA filter for SARS‐COV‐2 RNA found in an indoor space allowed to find an infected worker.

## 1 INTRODUCTION

On March 11, 2020, the World Health Organization (WHO) declared officially a global pandemic caused by coronavirus disease 2019 (COVID‐19), a highly transmittable and pathogenic viral infection caused by severe acute respiratory syndrome coronavirus 2 (SARS‐CoV‐2).^1^, ^2^

In Spain, schools and many nurseries re‐opened in September 2020 after 7 months of lockdown with the establishment of rigorous infection control practices, mainly by promoting frequent hand hygiene, guaranteeing periodic cleaning of surfaces, physical distance of at least 1.5 m, and increasing ventilation of the indoor spaces by opening windows and doors. In fact, the ventilation conditions during the first month of reopening schools in classrooms surveyed in Ciudad Real (Spain) were greatly better than those reported previously in the literature, being the median concentration of CO_2_ of 539 ppm for preschools and 565 ppm for primary classrooms.^3^ Moreover, the use of face masks was mandatory in indoor and outdoor spaces at school except for preschool children.^4^ Until June 23, 2021, a total of 260 699 children up to 9‐year‐old (around 7.5% of the total confirmed cases) were confirmed as SARS‐CoV‐2 positive in Spain.^5^

The COVID‐19 transmission is more likely indoors than outdoors which can be explained by airborne transmission through the presence of aerosol containing viable viral particles, generated by infected people.^6^ This especially occurs in closed and poorly ventilated spaces.^7^, ^8^, ^9^, ^10^, ^11^ Viable Delta variant SARS‐CoV‐2 has been detected in air collected by air samplers located ~3 m from an infected individual experiencing mild symptoms as well as through surface sampling of a mobile phone that was in frequent proximity to the individual's exhaled breath.^12^ The SARS‐CoV‐2 containing particles with diameters <100 μm can float in the air from minutes to several hours.^13^ For this reason, natural or mechanical ventilation is highly recommended in indoor environments to reduce the risks of virus transmission. However, natural ventilation depends on weather conditions and building structure, which make it difficult to achieve a high enough ventilation rate at all time. In those cases, the use of portable‐filter air cleaners (PACs) can be an effective supplementary measure to improve the cleaning of indoor air contaminated with virus‐containing aerosols. PACs are used to remove particulate matter from indoor air and a very common particulate filter is the high‐efficiency particulate air (HEPA) filter, in which the single‐pass efficiency of the filter media is >99.97% for 0.3 μm particles (Most penetrating particle size) with an equal or higher efficacy for other aerosol sizes.^14^ As measured by Liu et al., ^15^ the peak concentration of SARS‐CoV‐2 aerosols appears in two distinct size ranges, one between 0.25 and 1.0 μm, and other larger than 2.5 μm. The filtration efficiencies of HEPA filters, employed in most air purifiers is high enough to remove this size range of potentially infectious aerosols.

The analysis of dust collected on filters in central forced‐air heating, ventilation, and air conditioning (HVAC) or portable systems to determine the presence of biotic and abiotic contaminants in indoor air have been widely investigated and are collected in Haaland and Siegel's review.^16^ Several experimental studies have provided evidence for the potential of portable HEPA purifiers to eliminate airborne SARS‐CoV‐2.^17^, ^18^, ^19^, ^20^, ^21^ Rodriguez et al.^20^ analyzed the indoor air of positive patients in households before and after using a PAC. All the air samples collected (by using an MD8 Airport portable air collector with gelatin membrane filter) before using PACs were positive for SARS‐CoV‐2 while after switching on PACs, all samples except one were negative, but authors highlighted the fact that in the negative case the sampled room size was higher than recommended for the use of this device. In addition, the manufacturer of PAC used did not provide the clean air delivery rate (CADR), an important metric used to characterize air cleaners whose units are cubic meter per hour. On the contrary, Curtius et al.^22^ tested the efficiency and practicability of PACs in a high school classroom while regular classes were taking place. They monitored the aerosol number concentration for particles >3 nm at two locations in the room, the aerosol size distribution in the range from 10 nm to 10 μm and particulate matter PM_10_ with windows and door closed, concluding that the aerosol concentration was reduced by more than 90% within less than 30 min when running the PACs. Morris et al.^23^ detected SARS‐CoV‐2 in a COVID‐19 ward before activation of HEPA‐air filtration and also after filter deactivation but not during filter operation. SARS‐CoV‐2 RNA was evaluated in the various size fractions of air samples collected using National Institute for Occupational Safety and Health (NIOSH) BC 251 2‐stage cyclone aerosol samplers. Air filtration simulation experiments carried out by Ueki et al.^24^ quantitatively showed that an air cleaner equipped with a HEPA filter could continuously remove SARS‐CoV‐2 from the air.

During pandemic, HEPA filters have also been used as sampling system to test the presence of SARS‐CoV‐2.^19^, ^25^, ^26^ Burgos‐Ramos et al.^19^ analyzed HEPA filters exposed for 3 months in a waiting room and three treatment rooms from dental clinics. SARS‐CoV‐2 was detected in the filter of the waiting room but not in the treatment rooms. They concluded that the use of H_2_O_2_ solution (1%) for 1 min for mouth rinsing drastically reduced the possibility of SARS‐CoV‐2 spread during aerosol‐generating dental procedures. Recently, Zuniga‐Montanez et al.^25^ monitored the presence of SARS‐CoV‐2 RNA in five schools (96 classrooms) in Davis, California, by collecting weekly surface‐swab samples from classroom floors and portable HEPA units. Twenty‐two surfaces tested positive with qPCR. However, no confirmed COVID‐19 cases were identified among students associated with classrooms yielding positive environmental samples, concluding that the positive samples detected appeared to contain relic viral RNA.

Therefore, PACs with HEPA filters and an adequate CADR with respect to the room size are recommended as a supplementary and precautionary measure to reduce SARS‐CoV‐2 transmission, especially in indoor spaces, where many people are gathered such as classrooms. Additionally, PACs with HEPA filters are also recommended as a measure to improve indoor air quality.^27^, ^28^, ^29^, ^30^

Regarding the viral infection, there is strong evidence that respiratory transmission is dominant, with limited impact of transmission by surfaces.^31^ Nevertheless, surfaces can be used for viral monitoring due to the permanence of the virus on them, which can be useful as a surveillance tool.^32^, ^33^, ^34^ For example, the detection of SARS‐CoV‐2 RNA in air filters or by means of sponges on surfaces^35^ indicates the current or recent circulation of viruses in the space investigated, although the presence of viral RNA is not an indication of infectivity.

Based on these premises, the main objective of this study was to employ environmental monitoring of the presence of SARS‐CoV‐2 in HEPA filters and other surfaces in indoor spaces in which ventilation strategies are used to avoid virus transmission. The study was conducted for the selective screening in asymptomatic population groups in a school, a nursery, and a household of a social health center to identify and try to limit outbreaks at an early stage in these institutions of interest for Public Health. To approach this objective, a method for determining the presence of SARS‐CoV‐2 RNA in HEPA filters was developed, optimized, and validated under realistic conditions where PACs were running, and people infected with COVID were notified subsequently. Later, a monitoring campaign was conducted for 13 weeks in the three indoor environments.

## 2 MATERIALS AND METHODS

### 2.1 Portable Air Cleaners Characteristics and Operation

The tests were performed with commercially available PACs (Winix Zero Model AZBU330‐HWE and Winix Zero Pro Model AZPU370‐IWE). These air cleaners are equipped with True HEPA filters (H13) that remove 99.97% of the particles of 0.3 μm and according to the manufacturer, HEPA filters are certified with EN 1822:2019 Standard.^36^ The volume flow through the Winix Zero can be adjusted in five stages: sleep, low, medium, high, and turbo. According to the manufacturer the CADR for each stage is 60, 120, 196, 270, and 390 m^3^/h, respectively. The device is designed for rooms of up to 99 m^2^. In the case of Winix Zero Pro, it also presents five stages; sleep, low, medium, high, and turbo being the CADR 120, 122, 284, 330, and 470 m^3^/h, respectively, and it is designated for rooms of up to 120 m^2^. It is important to highlight that the room size specified by the manufacturer is a recommendation given to reduce the allergens or particulate matter from air and not to reduce SARS‐CoV‐2. Both PACs have been recommended to be used in stage “high” or “turbo” to reduce the risk of aerosol transmission, the CADR should be set as high as possible. The problem that presents the “turbo” mode is the noise and thus most of the time it is used at stage “high”. Table 1 summarizes the characteristics of operation of air cleaners used in this study. The PACs were running during the school lessons from the beginning to the end of lessons (about 5 h) every day, 5 days per week. In the nursery about 7 h per day, 5 days per week, while in the case of the household of the social health center, it was running continuously in the living room.

**TABLE 1:** Operation technical characteristics of air cleaners (Winix Zero and Winix Zero Pro) provided by manufacturer

Air Cleaner	Ventilation mode	CADR (m^3^/h)	Noise level (dB)	Power consumption (W)	Size (DxHxW) (cm)
Winix Zero	High	270	49	23	20.8 × 60 × 38
	Turbo	390	51	55	
Winix Zero Pro	High	330	40	21	24.5 × 60 × 41.5
	Turbo	470	55	87	

Regarding filtration characteristics, the air cleaners present a washable pre‐filter for coarse dust (made of fine mesh), an active charcoal filter for volatile organic compounds and the glass microfiber HEPA filter H13 for submicron and ultrafine particles. The air cleaners present a patented PlamaWave Technology® that produces OH radical with the aim of removing biological pathogens. However, due to OH radicals can initiate chemical oxidation indoors, leading to a wide variety of chemically complex and unhealthy products, this technology has been recommended to be deactivated.^37^, ^38^ Both PACs have UK allergy seal, ECARF (European Centre for Allergy Research Foundation) certified and AHAM (American Household Appliance Manufacturers) seal that refers to the certification of the CADR.

Regarding the installation of PACs, it is recommended to place them in the center of the room and when not possible at least at 1 m from the wall and far from the blackboard, windows or doors. The air intake flow of the PACs used is on the sides and front, and the air outlet flow is at the top. It has also been recommended to place them not directly on the floor but at 30 or 40 cm from it. The circulation of air must be free around the PAC. Due to the fact that PACs are used with children and disabled people (household) the safety of installation and operation must be guaranteed.

According to the national recommendation in classrooms to reduce risk in school, the recommended air changes per hour (ACH) was 5 or 6 h^−1^ for a classroom of 100 m^2^ with 25 students with 5–8 years old.^39^ This ACH can be reached with ventilation or with a combination of ventilation and filtration. Due to the high cost of buying tens of PACs for a school, most schools opted for buying only one PAC per classroom to supplement the ventilation, especially in winter in order to avoid having windows too open. Cross ventilation is recommended during pandemic. To know the ACH provided by air cleaners, the CADR must be divided by the classroom volume. If the classroom presents a volume of 140 m^3^ (surface 50 m^2^ multiplied by 2.8 m heigh) the ACH, taking into account the “high” mode of operation of PAC, would be 1.9 h^−1^ for Winix Zero and 2.4 h^−1^ for Winix Zero Pro (see Equation [1]). If PAC is running in “turbo” mode, then ACH is 2.8 and 3.4 h^−1^, respectively. The rest of ACH until 5 or 6 must be achieved by opening windows (ACH_ventilation_ = ACH_objective_‐ACH_aircleaner_).

(1) $$\mathrm{ACH}\;\left(h^{-1}\right)=\frac{\text{CADR}\;\left(m^{3}/h\right)}{V_{\text{room}}\;\left(m^{3}\right)}$$

### 2.2 Sponges

Surface samples were collected using Dry‐Sponges (3 M™ Dry‐Sponge; 3 M‐España, Spain, Madrid). These sponges were pre‐hydrated with 15 ml of an isotonic surfactant and virus‐inactivating liquid (P202130937) that allows to collect and preserve nucleic acids from surfaces and other substrates.^40^ On each room, sponges were gently rubbed over surfaces in contact with children's or people's hands and or over the gloves and clothing of the persons present. Sample sites in the household included the toilet supplies, fridge and microwave oven handles, and the main door handle. Sampling sites in the classrooms included surfaces such as tables, chairs, and entry door handles. The collected samples were stored under refrigerated conditions until processed in the laboratory.

### 2.3 Methodology for the determination of SARS‐CoV‐2 in HEPA filters and sponges

The procedure for the analysis of sponges in the laboratory has been described previously.^35^ Once in the laboratory, 2 ml of retained fluid was extracted from each sponge sample, collected in a screw cap tube, and centrifuged at 12 000 × g for 10 min. Viral RNA was extracted from 200 μl of solution taken from the bottom of the tube using the NucleoSpin RNA Virus kit (Macherey‐Nagel, Düren, Germany) according to the manufacturer's instructions.

The procedure for the analysis of HEPA filters included a wash of several sections of the filter (3 cm^2^ each one) located centrally and in the corners, placing each of them in a 15 ml adapted Amicon Ultra Centrifugal filters (Fisher Scientific SL, Spain) and adding 4 ml of the same solution used for hydrating the sponges. For the modification of the tubes, the internal flexible filter was removed leaving the inside rigid grid compartment, in which the filter section was placed. After 10 min of incubation at room temperature, the tubes were centrifuged at 4000 g for 5 min, collecting 2 ml of the solution and proceeding in the same way as previously explained for the analysis of sponges.

Detection of SARS‐CoV‐2 RNA was then performed by real‐time RT‐PCR using the designed FDA EUA 2019‐nCoV CDC kit (IDT, Belgium), based on N1 and N2 gene targets to detect SARS‐CoV‐2 and RNase P as an RNA extraction quality control.^41^ Amplifications with these three pairs of primers were set up at 45 cycles, following the manufacturer's indications. Real‐time RT‐PCR was carried out using the SuperScript III Platinum One‐Step qRT‐PCR Kit (Thermo Fisher, Massachusetts), according to manufacturer's protocol. Final volume of RT‐PCR reaction was 15 μl including 5 μl of RNA extraction. Nuclease‐free water was used as negative control. A CFX96 Touch Real‐Time PCR Detection System Thermal Cycler (BioRad, Berkeley) was used to carry out the reactions.

### 2.4 Design of the sampling campaign and measurement sites

The environmental surveillance of SAR‐CoV‐2 has been conducted in a school, a nursery, and a household of a socialhealth center in Ciudad Real (Spain). The main inclusion criteria to take part in the study was to be part of asymptomatic population groups without known active or recent positive cases. The sites included are:

- One classroom (8/9‐year‐old students with the obligation to wear a mask) of a school located in a rural area in the province of Ciudad Real (with 21 students and 6 teachers sharing space during the school day) considering that according to the protocols of Education and the school, they should not go to class with symptoms and if any student develops any suspicious symptoms, it is planned to isolate them immediately from the rest, outside the classroom. Moreover, rigorous infection control practices were applied, physical distance of at least 1.5 m and cross ventilation by opening windows and doors.
- Two rooms (with 11 and 16 children and two kindergarten aides in each) of a nursery in Ciudad Real (from 2 to 3 years of age without the obligation to wear a mask), with the same protocols as in the school.
- A household of a social health center (residence) in Ciudad Real (10 residents who live there with exemption from wearing a mask due to their disability and 9 assistants who share space with residents during their working hours). Rigorous infection control practices were also applied. Other 20 workers from the residence who enter to work in the household to be studied are nurses, psychologists, physiotherapists, educators, and cleaners. All workers must wear masks.

The characteristics of the indoor environments monitored are summarized in Table 2. These indoor environments had PACs with HEPA filters (Winix Zero Pro) as supplementary measure to natural ventilation. All spaces had cross ventilation. In the classroom of the school, the PAC was located at the center of the room and on a little table while in the nursery PACs were located on a side at 1 m from the wall and 1.5 m from the floor. In the household, the PAC was placed in the living room at 1 m from the wall and on the floor.

**TABLE 2:** Characteristics of indoor spaces monitored for the surveillance of SARS‐CoV‐2

Site	Number of people	Area / m^2^	Location of PAC	Hours of operation	Number of windows	Use of Masks	Cross ventilation
School classroom	27	43	Centre and on a little table	5 h a day/ 5 days a week	4	Yes	Yes
Nursery classroom A	13	25	On one side and on a table	~7 h a day/ 5 days a week	1^a^	No	Yes
Nursery classroom B	18	48	On one side and on a table	~7 h a day/ 5 days a week	2^a^	No	Yes
Household (Figure 4)	19	52	In living room on one side and on floor	24 h a day/ 7 days a week	2	No	Yes

^a^ number of balconies.

Before starting the monitoring campaign and to conduct this study, the approval of Drug Research Ethical Committee of the Integrated Care Management of Ciudad Real from SESCAM and the authorizations from Health, Educational and Social welfare provincial Administrations were needed. A document with detailed information of the study and an informed consent were sent to the centers to be signed by the children's and resident's legal guardians in order to perform the diagnostic tests for SARS‐CoV‐2 of that location for screening for asymptomatic patients infected by the virus.

The sampling period to detect environmental SARS‐CoV‐2 viral RNA was once a week for 13 weeks from February to May 2021. The samples (HEPA filters and surfaces) were collected every Friday, after the end of lessons in the case of the school and nursery, stored refrigerated, and processed in the laboratory the same day. When the detection of SARS‐CoV‐2 RNA in an indoor space was positive, a diagnostic test (PCR of nasopharyngeal exudate) to the individuals of that space for screening of asymptomatic patients infected by the virus was carried out. The diagnostic test was conducted by the nursing researchers and the microbiological analysis were carried out in the Microbiology Service of the General University Hospital of Ciudad Real. The positive cases detected must be reported to the competent Health Authorities since it is mandatory to declare them and to continue the procedure of epidemiological study of pertinent contacts, the surveillance and the follow‐up that is described in the protocols of Health, Social Welfare, and Education.

## 3 RESULTS AND DISCUSSION

### 3.1 Validation of the methodology in the HEPA filters

The validation of the methodology for the detection of SARS‐CoV‐2 RNA in the HEPA filters was carried out before starting the sampling campaign. On one hand, three PACs with HEPA filters running in two classrooms of different schools (school A and B) and the living room of a household of the social health center, were analyzed after knowing that there were people infected with COVID. Classrooms were closed for 2 weeks and in the case of the household, the infected residents were most part of time isolated in their bedrooms except those who were hospitalized. In these cases, the PACs were running for several weeks before the first positive individual was confirmed. The classrooms and the household were different from those included in the sampling campaign and described in section 2.4.

**FIGURE 1:**
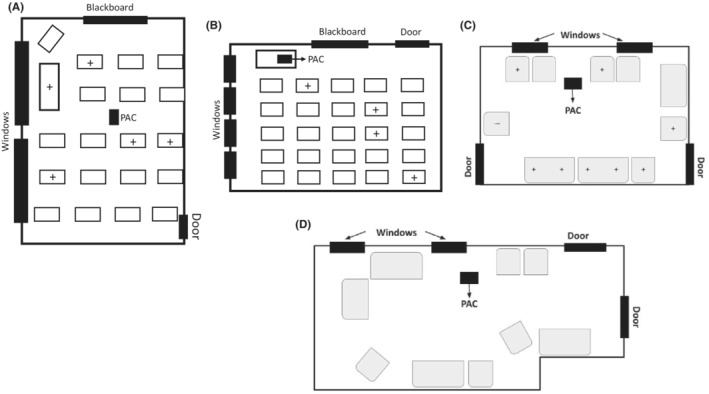
Diagrams of sampling sites. (A) Diagram of the classroom (53 m^2^) of school A (9/10‐year‐old students) showing the location of windows, doors, PAC (Winix Zero Pro), desks, and the positive confirmed cases (+). (B) Diagram of the classroom of school B (7/8‐year‐old students) showing the position of the PAC (Winix Zero) in the classroom (50 m^2^), desks, windows, and door and the position of the positive confirmed cases (+). (C) Diagram of the dining room (50 m^2^) of a household in the social health center with the location of the PAC (Winix Zero Pro), windows, doors, and the position of the 8 residents that tested positive (+). (D) Diagram of the dining room (52 m^2^) of the household in the social health center, that took part in the monitoring campaign, with the location of the PAC, windows, and doors.

The Ct values obtained in the SARS‐CoV‐2 RT‐PCR for the validation of the methodology in HEPA filters are summarized in Table 3. Figure 1A shows the position of the PAC in the classroom of school A, windows and door and the position of the positive confirmed cases by Health Authorities. This classroom is in a rural village at 6 km from Ciudad Real. It has 53 m^2^ with 2 big windows located on one side of the room. The windows and door were open during lessons and in addition, the classroom had mechanical ventilation according to the Spanish legislation for new buildings.^42^ There were 19 students (10 years old) and one teacher. The PAC (Winix Zero Pro) was placed almost in the center of the classroom on a little table. The number of people infected with COVID were 5 (4 students plus one teacher). On November 24, 2020, the classroom was closed (that was the last day the children attended lessons) and 6 days later, we collected the HEPA filter which was processed in the laboratory the following day. The analysis result was positive for SARS‐CoV‐2 RNA. Health Authorities did not consider that this outbreak took place in the classroom according to the epidemiological information provided by the children's families.

**TABLE 3:** Ct results obtained in the SARS‐CoV‐2 RT‐PCR for validation of the methodology for the detection of HEPA filters

Origin	Sample	Ct result range for PCR targeting N1 gene	Ct result range for PCR targeting N2 gene	Ct result range for PCR targeting Rp gene
Residence‐household with confirmed positive cases	Filter	34.59–38.19	37.18–38.17	34.43–36.87
	PCR Positive Control	30.78	33.59	30.87
School classroom 1 confined 2 weeks ago	Filter	38.86–39.99	40.65–43.30	37.26–38.12
	PCR Positive Control	30.30	34.28	31.39
School classroom 2 confined 2 weeks ago	Filter	38.25–39.11	37.03–39.84	36.37–38.02
	PCR Positive Control	29.33	32.21	29.31

Figure 1B shows the position of the PAC in the classroom of school B, windows, and door and the position of the positive confirmed cases by Health Authorities. This classroom is in a school from Ciudad Real. It has 50 m^2^ with 4 windows located on one side of the room. The windows and door were open during lessons, there were 25 students of 8 years old and one teacher. The PAC (Winix Zero) was placed on one side of the classroom near windows and on a table. Four students tested positive for COVID‐19. On December 15, 2020, the classroom was closed (that was the last day the children attended lessons), and 6 days later, HEPA filter was collected and processed in the laboratory. The analysis result was positive for SARS‐CoV‐2 RNA even after 7 days have elapsed since the occupation of the classroom. As before, Health Authorities did not consider that this outbreak took place in the classroom according to the epidemiological information provided by the children's families. However, since the virus was found in the filters seems likely that the infections in fact occurred in the classrooms, especially in school A where 5 positive people including the teacher tested positive.

After the start‐up of the PACs in a household from the social health center in September 2020 (where 9 residents live there and 9 assistants share space during their working hours) an outbreak began on October 7, 2020, infecting a total of 6 auxiliaries and 8 residents. The infected assistants were on sick leave until epidemiological and clinical discharge. The positive residents remained in their home but almost did not share common spaces (such as the living room where the HEPA filter was), each one stayed most of the time in their room, except the three residents who required hospitalization, some still had a positive PCR (although was already discharged epidemiologically according to Health protocols) in the days prior to the collection of samples. In this indoor environment not only the analysis of the HEPA filter was conducted but also two surface samples (e.g., armrests of armchairs, tables, pens, hair or toothbrush handles, doorknobs, switches, household appliances, hangers) were collected with sponges. Filter PCR analysis was performed on November 23, 2020, and was positive. In the residence, the surfaces are cleaned in each 8‐h shift at least twice. Surface samples were negative for SARS‐CoV‐2 RNA. Figure 1C show the location of the PAC in the common room on one side of the room, on the floor and near the windows that remained open during the time they shared in such space.

It is important to highlight that despite the cross ventilation in all indoor environments (in one classroom even with mechanical ventilation), the use of masks by children in the schools and the proximity of some PACs to the window, all HEPA filters trapped the virus and tested positive for SARS‐CoV‐2 RNA.

On the contrary, one blank sample (an HEPA filter new without having been exposed to indoor air) for each PAC model, was processed in the laboratory in the same way as sampled HEPA filters. Blanks were negative for SARS‐CoV‐2 RNA by PCR.

### 3.2 Environmental surveillance of SARS‐CoV‐2 RNA in indoor environments

Before starting the environmental surveillance campaign, the HEPA filters were changed in all the studied places, as soon as all the permits were received from the competent authorities.

The results obtained during the study for the surveillance in the classrooms of the nursery and the school, described in section 2.4, were negative for SARS‐CoV‐2 RNA, both for the HEPA filters and surfaces, for the whole period. During this period, no student or teacher was reported with COVID‐19, which agrees with the negative results for SARS‐CoV‐2 RNA found in HEPA filters. In the case of the household of the social health center with 10 residents and 9 assistants, the result of the analysis in the first week (February 19, 2020) showed a negative result from sponges and a positive result from the HEPA filter (Ct values in the PCR of the filter for the N1, N2, and Rp genes were 40.03, 38.69, and 39.03, respectively) (Figure 1D). Therefore, all the residents and assistants who shared air in this household were individually tested, in total 35 people. These analyses detected only a positive nasopharyngeal PCR. This was a worker who started working the week in which the study began after finishing his sick leave and having had a COVID‐19 infection. All the rest of assistants and the residents were negatives for the PCR screening. The worker was diagnosed while on vacation on January 12, 2021, and therefore, it was not an outbreak in the residence. When his clinic improved, he tested again positive on February 1 and a little after a week he had positive serology for SARS‐CoV‐2 and obtained epidemiological and occupational discharge (Health Administration published a new protocol on January 1, 2021, indicating that it was not necessary to have negative PCR for health or social health professionals for their incorporation to work, meeting other requirements). When the sampling campaign began, the house met inclusion criteria (such as not having active or recent cases) but the researchers were unaware about the incorporation of this worker to the household under study. The positive worker reported that he had only removed his mask for drinking water. The positive result from the HEPA filter indicates a case of relic SARS‐CoV‐2 RNA as reported by Zuniga‐Motanez et al.^25^ in the environmental monitoring of schools using portable HEPA filtration units. For that, we established at the beginning of the monitoring campaign the inclusion criteria. Our intention was to detect relatively fresh viral RNA (between 1 and 5 or 7 days) what may be considered among other factors for possible interventions in the sampling site.The result in the following week of the study (second in the sampling period) showed only a positive result for the sponge, indicating presence of the coronavirus on the surfaces of the household (Ct values in the PCR of the sponge for the N1, N2, and Rp genes were 35.64, 33.82, and 35.00, respectively). Notably, once the results were known, the worker exercised extreme caution and avoided removing the mask during the work period, which could explain the non‐detection of the virus in the filter after that week. It is necessary to take into consideration that viral detection from the sponges could be affected by the regular cleaning carried out mainly with bleach in these spaces. From the third week on, all the environmental PCR results were negative. At the residence, the vaccine against SARS‐CoV‐2 was completed on February 11, 2021. During this period, nobody (residents or workers) notified to have had COVID‐19 what would agree with the negative results for SARS‐CoV‐2 RNA found in HEPA filters in the living room of the household.

It is important to consider that the individual excretion and the viral concentration in the studied environment are not known, nor is it known how fragmented the genetic material of viruses is once they begin to degrade in the environment, these factors would interfere with subsequent molecular detection. The results of this study, in which the molecular analysis were carried out weekly, show that the viral RNA could be detected in the HEPA filter with a single positive individual; despite not knowing the excretion level of this infected individual, it was found that there was no contagion among the people who shared the room, therefore, it was not enough viral excretion to generate contagion in this case, but it was enough to be detected in the filter.

The possibility of detecting the SARS‐CoV‐2 virus RNA in HEPA filters housed in PACs could help to track in which room and even when SARS‐CoV‐2 virus was present, and therefore, the most probable place and time of transmission could potentially be traced.

It is important to mention that the monitoring campaign started as soon as all permits and authorizations were received from the competent authorities, but it was at a time of the pandemic when the third wave, after Christmas, has already passed with a cumulative incidence in the region of 41 cases per 100 000 inhabitants.^43^

## 4 CONCLUSIONS

The studies conducted up to date have measured SARS‐CoV‐2 in rooms (mainly hospitals but also in nonhealthcare environments) where people were already infected with SARS‐CoV‐2. By contrast, herein we investigated the presence of SARS‐CoV‐2 in HEPA filters housed in PACs running in places of great interest for public health with apparently healthy people before anyone had symptoms or tested positive with the aim of trying to prevent a possible outbreak.

A methodology for the detection and surveillance of SARS‐CoV‐2 in HEPA filters of PACs was developed and validated in this study. The fact that all HEPA filters tested negative in the classrooms along the whole study agrees with the fact that students or teachers were not notified to have had COVID‐19 in that period. In the case of the social health center, only one HEPA filter tested positive which agrees with the fact that one worker tested positive after a nasopharyngeal SARS‐CoV‐2 PCR test. Despite the use of masks and the fact that the worker recovered from the COVID‐19 disease, the HEPA filter trapped the SARS‐CoV‐2. The results of this study support that HEPA filters concentrate and inactivate the coronavirus while providing a tool for SARS‐CoV‐2 surveillance. The weekly analysis enables an early detection, although when the viral RNA is detected in the filter the transmission risk has already begun to occur, this approach could be used for the early detection of virus circulation with the possibility to implement measures to reduce virus transmission risks and the disease incidence.

Finally, due to the paper use portable air cleaners with HEPA filters for monitoring of pathogens in the air, future research could be focused on the comparison between the described method in this study and the current air sampling methods.

## AUTHOR CONTRIBUTIONS

Isabel G. Fernández de Mera involved in sample collection and methodology. Carmen Granda and Florentina Villanueva involved in conceptualization, sampling campaign, and funding acquisition. Marta Sánchez‐Sánchez and Alberto Moraga‐Fernández performed analysis of HEPA filters and sponges. Christian Gortázar and José de la Fuente contributed in supervision and investigation. All authors involved in original draft, reviewing, and editing the manuscript.

## CONFLICT OF INTEREST

The authors declare that they have no known competing financial interests or personal relationships that could have appeared to influence the work reported in this paper.

## ETHICS STATEMENT

The study has the definitive approval of the Drug Research Ethical Committee of the Integrated Care Management of Ciudad Real from SESCAM dated January 26, 2021, Act 01/2021. We did not use individual patient data and did not perform animal sampling. The basic ethical principles of non‐maleficence, justice, autonomy, and beneficence have always been respected and it was carried out following the norms of Good Clinical Practice and the ethical norms of the Declaration of Helsinki, promulgated by the World Medical Association in June of 1964 and subsequent modifications or the European Convention on Human Rights and Biomedicine, of April 1997 (Oviedo Convention). Intimacy, privacy, confidentiality, and the protection of patient data were also respected at all times.

Regarding observational studies: Order SAS / 3470/2009, of December 16, which publishes the guidelines on observational post‐authorization studies for medicines for human use and Order of 09/21/2010 of the Ministry of Health and Social Welfare, which regulates observational post‐authorization studies with medicines for human use in Castilla‐La Mancha. Regarding biomedical research that involves the use of human biological samples: Law 14/2007, of July 3, on biomedical research and Royal Decree 1716/2011, of November 18, which establishes the basic authorization requirements. and operation of Biobanks for biomedical research purposes and the treatment of biological samples of human origin, and the operation and organization of the National Registry of Biobanks for biomedical research is regulated.

The results were recorded in a computerized database for research purposes of the network unit of the Castilla La Mancha Health Service (SESCAM), with a secure connection (restricted and private access with personal professional password for use and editing of data) guaranteeing the confidentiality of the data, with backup copy, where the data are kept.

## Data Availability

The data that support the findings of this study are available from the corresponding author upon reasonable request.
